# Is conventional transbronchial lung biopsy out: The evaluation of clinical value in diffuse parenchymal lung disease

**DOI:** 10.1111/crj.13524

**Published:** 2022-08-12

**Authors:** Lefei Zhou, Feng Wang, Xiaoguang Xu, Lili Xu, Zhen Wang, Zhaohui Tong

**Affiliations:** ^1^ Department of respiratory and Critical Care Medicine, Beijing Institute of Respiratory Medicine and Beijing Chao‐Yang Hospital Capital Medical University Beijing China; ^2^ Department of respiratory and Critical Care Medicine, Ordos Central Hospital, Ordos Clinical Medical College of Inner Mongolia Medical University Inner Mongolia China; ^3^ Department of respiratory and Critical Care Medicine Affiliated Hospital of Jilin Medical University Jilin China

**Keywords:** diffuse parenchymal lung disease, DPLD, TBLB, transbronchial lung biopsy

## Abstract

**Introduction:**

Transbronchial lung biopsy (TBLB) is a relatively safe technique routinely employed by pulmonologists for the diagnosis of diffuse parenchymal lung disease (DPLD). Cryobiopsy is associated with higher diagnostic yield and a favorable risk/benefit ratio. Nevertheless, TBLB remains the representative method for definite diagnosis in developing countries.

**Objectives:**

This study aimed to evaluate whether the results obtained from TBLB had clinical value to pulmonologists in the management of DPLD.

**Methods:**

We performed a retrospective analysis of patients who underwent conventional TBLB for the diagnosis of DPLD from May 1, 2017, to April 30, 2019, at the Beijing Chao‐yang Hospital, Capital Medical University. The clinical value of TBLB was defined as leading to a specific histopathological diagnosis or being consistent with the clinical and radiological data.

**Results:**

Seven hundred and forty‐three patients with suspected DPLD were recruited. Conventional TBLB was considered clinically valuable in 439 procedures (59.1%), including 360 cases provided with definitive histopathological diagnoses, and 79 cases that were consistent with the working diagnoses. Among the 439 cases of clinically valuable TBLBs, 88 (20.0%), 37, 77 (10.7%), and 61 (13.9%) cases were diagnosed as connective tissue disease‐related interstitial lung disease, definite histopathological diagnoses, malignancies, and nonspecific interstitial pneumonia, respectively.

**Conclusions:**

Conventional TBLB served as a key determinant or provided supplementary information in the final diagnosis of non‐infectious DPLDs. TBLB decision‐making should therefore be based on clinical and radiological data.

## Graphical abstract

This retrospective study on transbronchial biopsies in pulmonary infiltrative diseases collected 743 patients. In this series, transbronchial lung biopsy (TBLB) were useful in 59% of cases, giving either a true diagnosis or consistent with the working diagnosis. Conclusion that conventional TBLB served as a key determinant or provided supplementary information in the final diagnosis of non‐infectious diffuse parenchymal lung diseases (DPLDs). TBLB decision‐making should therefore be based on clinical and radiological data.

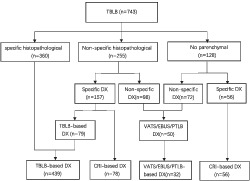

## 1 INTRODUCTION

Conventional transbronchial lung biopsy (TBLB) is a relatively safe technique routinely employed by pulmonologists for the diagnosis of diffuse parenchymal lung disease (DPLD). Biopsies of the lung have been obtained via bronchoscopy for nearly 60 years.^1^ In routine practice, both clinical evaluation and pattern recognition from a high‐resolution computed tomography (HRCT) scan of the thorax is not always helpful in the diagnosis of DPLD. When clinical‐radiological information discordance occurs in DPLD, biopsies may be necessary. Cryobiopsy is associated with a higher diagnostic yield and a favorable risk/benefit ratio.^2^ Nevertheless, TBLB remains the representative method for definite diagnosis in developing countries. Previous studies^3^, ^4^, ^5^ have mostly focused on the diagnostic accuracy of TBLB in terms of histology, as well as on the complications of TBLB procedures in various patient populations. Therefore, the present study aimed to evaluate whether the results obtained from TBLB had clinical value to pulmonologists in the management of DPLD.

## 2 MATERIALS AND METHODS

We reviewed our database of patients who underwent TBLB at the Beijing Chao‐yang Hospital, Capital Medical University from May 1, 2017, to April 30, 2019. All subjects authorized the review of their clinical records and informed written consent was obtained before the procedures. The ethics committee of the Capital Medical University approved the study.

### 2.1 Inclusion criteria and exclusion criteria

The indication for TBLB was obtained from the bronchoscopy report. TBLBs of a lung mass, solitary nodule, or lung cavity were excluded. The computed tomography (CT) patterns were reviewed by two pulmonologists, and those without bilateral multifocal parenchymal changes were excluded. Cases whose final diagnosis was an infection were also excluded. All subjects who were suspected of DPLD and underwent TBLB to obtain adequate parenchymal lung tissue for meaningful histological analysis were enrolled.

### 2.2 Subjects

The recorded clinical information for all patients included basic clinical characteristics, spirometry results, high‐resolution computed tomography (HRCT) features, bronchoalveolar lavage fluid (BALF) number counts, anesthesia mode, and complications.

TBLB was performed by a group of dedicated and experienced pulmonologists using a flexible bronchoscope (Olympus BF LUCERA, Tokyo, Japan) and was carried out under topical or general anesthesia (using propofol and remifentanil). TBLB was taken using standard biopsy forceps (Micro‐Tech MTN‐BF‐18/12‐A), targeted to the appropriate lung segments wherein areas of abnormality were seen on HRCT. Samples were obtained from one or multiple sites, depending on the radiological pattern and distribution of the disease in a single lung. Imprint smears of the specimens were not prepared to prevent crushing artifacts, and the specimens were instead directly transferred into a fixative. Other bronchoscopy procedures, such as bronchoalveolar lavage (BAL), culture of the lower respiratory tract secretions, and brush sampling of the lesion, were simultaneously performed. Patients were observed and were instructed to immediately report any aggravation of symptoms. A check chest radiograph was done post‐procedure only if the patient complained of pain and if discrepancy in breath sounds was observed.

The clinical value of TBLB was defined as a specific histopathological diagnosis and that was consistent with the working diagnosis based on clinical information and radiological data. The TBLB was regarded as clinically not valuable if the procedure failed to obtain the lung tissue, the tissue amount was not adequate to reach the pathological diagnosis, and if the final pathological diagnosis was still non‐specific. TBLB was considered as non‐specific histopathologically when it lacked the criteria sufficient to define a characteristic histopathological pattern. Samples that were considered inadequate (e.g., too small or airway wall with no alveoli lung parenchyma) were non‐parenchymal.

### 2.3 Statistical analyses

Statistical analyses were performed using SPSS 23.0 (IBM, NY, USA) with data represented as means for continuous variables and percentages for categorical variables. The comparisons were performed using the unpaired *t* test and chi‐squared test, as appropriate. *P* < 0.05 was considered statistically significant.

## 3 RESULTS

**FIGURE 1:**
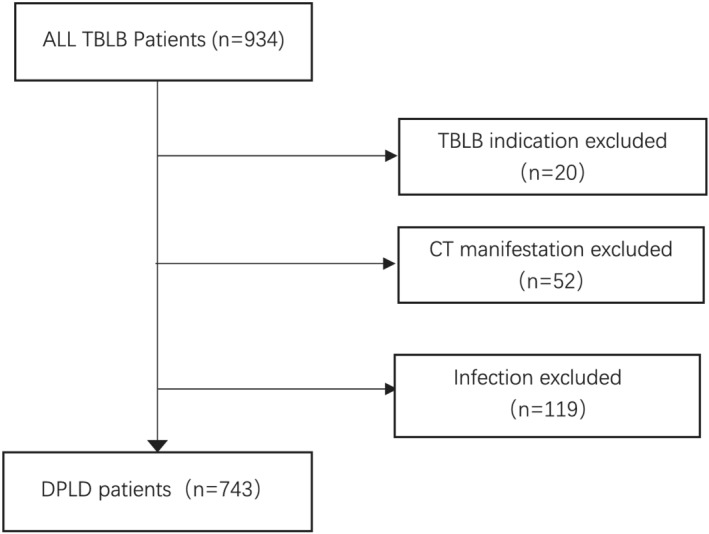
The flow chart depicting patient selection for the study. DPLD, diffuse parenchymal lung disease; TBLB, transbronchial lung biopsy

During the 2‐year study period, a total of 934 patients underwent TBLB. Based on the inclusion and exclusion criteria, 743 patients were enrolled in the study (Figure 1). During this period, 4100 bronchoscopies were carried out at the Beijing Chao‐yang Hospital. The subjects' characteristics are summarized in Table 1. There were 391 (52.6%) males and 352 (47.4%) female patients with a mean age of 58.2 (20–87) years. The spirometry results were as follows: force expiratory volume in 1 s (FEV1): 2.18 L (0.68–4.8 L); FEV1%: 84.59% (57.2%–94.8%); forced vital capacity (FVC): 2.75 L (0.7–6.06 L); FVC%: 87.6% (32.8%–97.2%); diffusing capacity for carbon monoxide (DLCO): 5 L (1.17–13.73 L); DLCO%: 69.79% (15.9%–89.3%). Topical anesthesia was used in 619 (83.3%) patients. The biopsy area was mainly from the basal right lung. Four to eight samples were taken from one or multiple sites. The TBLB complications include pneumothorax and bleeding. In this study, 25 (3.3%) patients had pneumothorax; only five needed management with intercostal drainage, and the tubes were removed in less than 4 days. The others recovered through oxygen therapy. Seven patients had mild (5) and moderate (2) bleeding, which was controlled with conservative bronchoscopic treatment.

**TABLE 1:** Clinical characteristics and complications in patients who underwent transbronchial lung biopsy (TBLB)

Patient characteristic (*n* = 743)	No. (% or SD)
Median age (SD), *y*	58.2 (11.8)
Male, No. (%)	391 (52.6)
Topic anesthesia (%)	619 (83.3)
Biopsy position‐right basal lung, (%)	447 (60.0)
Biopsy No, (SD)	4 (2)
Pneumothorax, No. (%)	25 (3.3)
Drained Pneumothorax, No. (%)	2
Mild bleeding, No. (%)	5
Moderate bleeding, No. (%)	2
Severe bleeding, No, (%)	0
FVC	2.75 (0.89)
FVC%	87.6 (21.41)
DLCO	5.0 (1.89)
DLCO%	69.79 (19.86)
FEV1	2.18 (0.72)
FEV1%	84.59 (20.47)
FEV1/FVC	79.06 (8.45)

Abbreviations: DLCO, diffuse capacity of the lungs for carbon monoxide; FEV1, forced expiratory volume in 1 s; FVC, forced vital capacity.

The TBLB results were considered clinically valuable (definitive histopathological diagnoses) in 360 (48.5%) out of the 743 procedures. Of these from non‐histopathological diagnoses, 157 provided specific histopathological diagnoses, and 79 (10.6% of all TBLB) were valuable in excluding certain disorders, which were consistent with the working diagnosis and the radiological data as TBLB‐based diagnosis. Therefore, a total of 439 (59.1%) TBLB results were clinically valuable out of 743 procedures. If the 128 cases that did not obtain parenchymal lung disease were excluded, the final value would be 439 (71.4%) out of 615 procedures. Another 78 (10.4% of all TBLB) completely relied on the BALF test and follow‐up clinical data and were classified as clinical‐radiological information‐based (CRI‐based) diagnoses.

**FIGURE 2:**
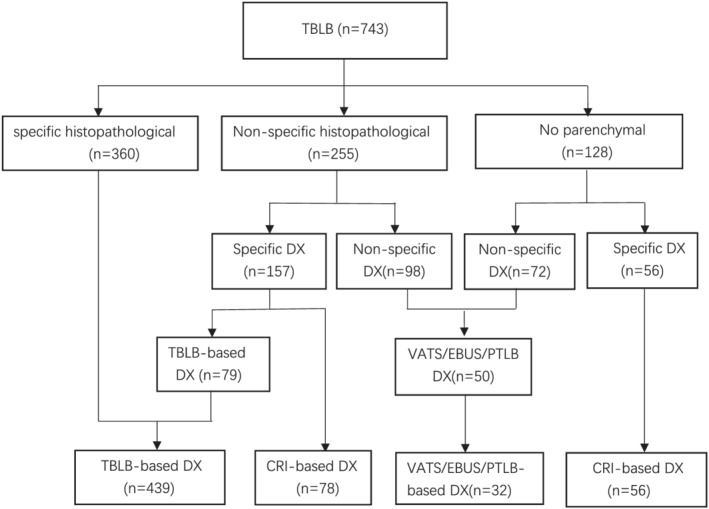
The classification and clinical utility of TBLB in the diagnosis of diffuse parenchymal lung diseases. CRI‐based, clinical and radiological information‐based; DX, diagnosis; PTLB, percutaneous lung biopsy; SLB, surgical lung biopsy; TBLB, transbronchial lung biopsy; TBLB‐based, transbronchial lung biopsy‐based

The remaining 304 (40.9%) TBLB results were considered clinically not valuable, which included the 128 (17.2% of all TBLBs) cases that obtained no parenchymal lung tissue and 176 (69.0% of all non‐specific histopathological TBLBs) that lacked diagnostic histopathological characteristics. When TBLB results were considered not valuable, video‐assisted thoracic surgery (VATS), endobronchial ultrasound‐guided transbronchial needle aspiration (EBUS‐TBNA), and percutaneous transthoracic needle biopsy (PTNB) were performed in 50 (14, 13, and 22, respectively) out of 248 patients. Of them, 32 (64.0% of 50) had definite diagnoses. Definite diagnosis was also identified in 57 (18.7% out of 304) patients using test samples from BAL and the specimen brush. Among the 128 patients in whom no parenchymal tissue has been collected, the specific diagnoses were established in 56 (43.75%) patients using the follow‐up clinical data that was based on the progression of clinical condition in conjunction with HRCT manifestations and were also classified as CRI‐based diagnoses (Figure 2). From this group, 21 were diagnosed as having idiopathic pulmonary fibrosis (IPF).

The distribution of TBLB‐based specific diagnoses and CRI‐based clinical diagnoses are shown in Table 2. Among the 439 cases of clinically valuable TBLBs, 88 cases (20.0%) were diagnosed as connective tissue disease‐related interstitial lung disease (CTD‐ILD), 37 cases were identified by TBLB as definite histopathological diagnoses, 77 cases (10.7%) were malignancies, and 61 cases (13.9%) were nonspecific interstitial pneumonia. Among the 134 CRI‐based specific diagnoses, the majority were IPF (47 cases), 17 cases were identified as definite histopathological diagnoses, 37 cases were hypersensitive pneumonia, and 34 cases were CTD‐ILD.

**TABLE 2:** The distribution of specific diagnoses based on TBLB and clinical‐radiological data

Diagnosis	TBLB‐based	CRI‐based	Total
CTD‐ILD	88	34	122
Malignancy	77	0	77
NSIP	61	0	61
HP	55	37	92
IPF	47	41	88
COP	44	0	44
SAR	43	7	50
CEP	6	6	12
RB‐ILD	6	0	6
AFOP	5	0	5
AAV	4	7	11
PAP	2	0	2
DAD	1	0	1
PLCH	0	2	2
Total	439	134	573

Abbreviations: AAV, ANCA‐associated vasculitis; AFOP, acute fibrinous organizing pneumonia; CEP, chronic eosinophilic pneumonia; COP, cryptogenic organizing pneumonia; CRI‐based, clinical and radiological information‐based; CTD‐ILD, connective tissue disease‐related interstitial lung disease; DAD, diffuse alveolar damage; HP, hypersensitive pneumonia; IPF, idiopathic pulmonary fibrosis; NSIP, nonspecific interstitial pneumonia; PAP, pulmonary alveolar proteinosis; PLCH, pulmonary Langerhans cell histiocytosis; RB‐ILD, respiratory bronchiolitis‐associated interstitial lung disease; SAR, sarcoidosis; TBLB‐based, transbronchial lung biopsy based.

The clinical characteristics, spirometry, BALF cell count, and radiological features were compared between TBLB‐based IPF and CRI‐based IPF, and no significant differences were observed. The most common features in patients with IPF were subpleural predominance (56.0%), basal predominance (55.0%), reticular abnormality (75.0%), and honeycombing (56.0%) (Table 3).

**TABLE 3:** Differences between TBLB‐based and clinical‐radiological information‐based IPF

	TBLB‐based (*n* = 47)	CRI‐based (*n* = 41)	Total	*P* value
Age	61.55 ± 7.01	62.59 ± 9.53		0.55
Male/Female	40/7 (85/15)	28/13(68/32)		0.07
FVC	2.78 ± 0.84	3.00 ± 0.76		0.21
FVC%	37.91 ± 9.44	41.13 ± 6.38		0.48
DLCO	5.04 ± 1.59	4.57 ± 1.57		0.20
DLCO%	51.69 ± 17.2	49.85 ± 18.48		0.66
FEV1	2.21 ± 0.63	2.28 ± 0.64		0.37
FEV1/FVC	78.03 ± 14.86	83.00 ± 6.06		0.11
BALF, N%	58.96 ± 12.74	53.76 ± 15.06		0.18
BALF, S%	2.5 ± 1.31	3.88 ± 1.73		0.50
BALF, L%	29.57 ± 10.90	30.68 ± 13.38		0.74
Upper predominance	5 (10.6)	3 (7.3)	8 (9.0)	0.36
Basal predominance	19 (40.4)	36 (87.8)	55 (62.5)	0.08
Subpleural predominance	22 (46.8)	34 (82.9)	56 (63.6)	0.72
Ground glass opacity	17 (36.2)	20 (48.8)	37 (42.0)	0.40
Reticular abnormality	41 (87.2)	34 (82.9)	75 (85.2)	0.49
Honeycomb	29 (61.7)	27 (65.8)	56 (63.6)	0.73
Fibrotic streaks	23 (48.9)	14 (34.1)	37 (42.0)	0.62
Traction bronchiectasis	17 (36.2)	11 (26.8)	28 (31.8)	0.91
Nodule	11 (23.4)	7 (17.1)	28 (31.8)	0.38
Consolidation	4 (8.5)	1 (2.4)	5 (5.6)	0.23
Thin‐walled translucent shadow	7 (14.9)	10 (24.3)	17 (19.3)	0.65

Abbreviations: BALF, L%, percentage of lymphocyte in BALF; BALF, N%, percentage of neutrophils in BALF; BALF, S%, percentage of eosinophils in BALF; CRI, clinical and radiological information; DLCO, diffusing capacity of lungs for carbon monoxide; FEV1, forced expiratory volume in 1 s; FVC, forced vital capacity; IPF, idiopathic pulmonary fibrosis; TBLB, transbronchial lung biopsy.

Malignancy was ultimately diagnosed in 83 (11.2%) patients out of the 743 enrolled patients. In 77 out of these 83 (92.3%) patients, the diagnosis was provided by TBLB. Primary lung cancer and metastatic malignancy were diagnosed in 57 (7.7%) and 20 (2.7%) out of the 743 patients, respectively. Of them, 22 (28.6%) had primary pulmonary mucinous adenocarcinoma, with 100% of the diagnosis provided by TBLB. The diagnostic rate of TBLB was 74.0% (five diagnoses by VATS) and 71.4% (one approved by PTNB) for primary lung cancer and metastatic malignancy, respectively.

Of all TBLB procedures, pneumothorax and bleeding occurred in 25 (3.3%) and 7 out of 743 patients, respectively. No death occurred during the study period.

## 4 DISCUSSION

DPLD currently remains one of the most challenging medical conditions in terms of both diagnosis and treatment. Without a correct diagnosis, the prognosis may be inaccurate, and curative therapy might not be administered. Therefore, differentiating the various DPLDs is paramount and urgently warranted. In some cases, the term DPLD is used to describe bilateral multi‐lobar parenchymal changes. However, the present study only included non‐infectious ill‐defined radiographic lesions, and hence, a pathological process can be diffuse in a localized area of the pulmonary parenchyma. The specimens were obtained from non‐infectious parenchymal lung tissue as mentioned previously. In this present study, patients underwent HRCT evaluation prior to bronchoscopy to guide the TBLB performed by an experienced pulmonologist. Wells et al. reported that pattern recognition from an HRCT scan of the thorax is helpful in some DPLD diseases.^6^, ^7^ TBLB, also known as transbronchoscopic or transbronchial lung biopsy, refers to the bronchoscopic technique of obtaining lung parenchymal tissue for histological analysis. The first TBLB was described as the rigid bronchoscope technique and was performed in 13 patients by Andersen et al. at the Mayo Clinic in 1965.^8^ Among the subsequent studies, 84% of 450 TBLB cases were obtained using a rigid bronchoscope.^9^ A decade later, Levin et al. reported their experience in using a flexible bronchoscopic transbronchial biopsy,^10^ which increased the popularity of the technique and demonstrated that TBLB could be obtained with minimal mortality and morbidity.^4^, ^11^, ^12^, ^13^, ^14^ When the TBLB technique is used in clinical practice, a positive biopsy is defined as that having diagnostic histology, histology that supported a diagnosis, or histology consistent with the final diagnosis.^4^ Most, if not all, of the published studies have focused on the diagnostic accuracy of TBLB histological analysis in patients with diffuse lung diseases.^3^ In contrast to earlier reports on TBLB, the present study focused on how TBLB results are used by clinicians. The main objective was to assess whether the routinely performed TBLB was clinically useful and necessary in the management of patients with DPLD. The results of the present study indicated that TBLB is clinically useful in 59.1% of cases, which was consistent with the overall TBLB diagnostic yield of around 25%–75%.^3^, ^15^, ^16^, ^17^ Ensminger and Parkash have evaluated whether the results of TBLB were clinically useful in the management of patients with diffuse pulmonary disorders and indicated that TBLB was a clinically useful test in approximately 75% of all procedures.^3^ Their study was under fluoroscopic guidance and they had included infectious disease. Infection was the most common indication for TBLB in some studies, and for cases of infection occurring as infiltrative lung disease, studies reported a TBLB diagnostic yield of 38%–82%.^16^, ^18^ In another report with 664 enrolled patients, TBLB was considered clinically helpful in 202 (30.4%) patients, including 114 cases wherein definitive histopathological diagnoses were provided and 88 cases that were consistent with the working diagnoses; this study also concentrated on a relatively narrow spectrum of DPLD, such as idiopathic interstitial pneumonia (IIP), CTD‐ILD, and pulmonary alveolar proteinosis (PAP).^19^

Excluding the 128 non‐parenchymal cases, the final number of useful TBLB in our study is 439 (71.4%) out of 615, indicating that sophisticated technique and appropriate patient selection may increase the diagnostic power of TBLB. In addition, BALF and the specimen brushing technique can be simultaneously performed with TBLB. From the data of this present study, 57 out of 304 patients in whom the TBLB was initially considered non‐useful, benefited from BALF and specimen brushing. We encourage performing BALF and specimen brushing with TBLB. The reason is that it may result in an occult diagnosis even without specific TBLB results. For example, if BALF CD1a is positive >5% or if Birbeck granules are identified in macrophages, it is highly suggestive of Langerhans cell histocytosis.^20^

CTD‐ILD is the most common type of DPLD, and its histological patterns are different and largely dependent on the underlying CTD. Therefore, it can manifest as fibrotic ILD or infiltrative ILD. The histological entities of IIP can also apply to patients with CTD‐ILD.^21^ In the present study, 42.0% of CTD‐ILD were TBLB‐based diagnoses, which may have been due to our patients not having specific symptoms, or largely due to the serum level of auto‐antibodies. Moreover, our patients may also have been on the stage of acute exacerbation and histologically be characterized as infiltrative, which is more commonly obtained by TBLB than the fibrotic pattern. Histopathological analysis may play an important role in diagnostic decision‐making or the prognostic evaluation of CTD‐ILD,^22^, ^23^ because evidence‐based procedures are necessary for clinical practice, especially since most patients with CTD‐ILD may need corticosteroid treatment. Therefore, our study also encourages the exploration of the correlation between histopathological patterns and disease prognosis and diagnosis.

In our study, 47 (53.4%) out of all 88 cases were TBLB‐based IPF. TBLB was viewed specifically as a diagnostic method and was consistent with the CRI‐based diagnosis in the remaining cases. Moreover, the comparison between TBLB‐ and CRI‐based IPF cases revealed no significant difference in terms of basic characteristics, spirometry, BALF number count, and HRCT features, with reticular abnormalities and honeycombing being the most common, and upper distribution and nodules being the rarest manifestations. Our study may suggest features that are atypical of IPF. Experimental treatment with close follow‐up may be helpful in the decision‐making process regarding patient management, and cryobiopsy may be a good substitute if the final diagnosis remains uncertain.^2^ The latest guidelines pertinent to the diagnosis and management of IPF included a weak recommendation, with low‐quality evidence against using TBLB in the evaluation of IPF in the majority of patients.^24^ Raghu et al. previously performed a retrospective analysis of patients with proven usual interstitial pneumonia (UIP). In their study, TBLB specimens were diagnostic of UIP in 7 out of 22 patients.^25^ In another study by Tomassetti et al., TBLB detected UIP in 30% of the cases, with high specificity and positive predictive value, but low negative predictive value.^26^ Recently, a study noted that TBLB combined with clinical and HRCT data can lead to a confident and accurate diagnosis in 20%–30% of patients with fibrotic ILD.^6^ Despite the lack of prospective studies investigating the definite role of TBLB in IPF, and despite its poor sensitivity, TBLB might be a reasonable option for patients with suspected fibrotic ILD, who cannot be diagnosed based on clinical‐radiological information alone, or who cannot tolerate transbronchial lung cryobiopsy (TBLC) or surgical lung biopsy (SLB).

For malignancies in the present study, 100% of the diagnosis have been provided through TBLB in patients with patchy or widespread diffuse parenchymal disease, especially primary pulmonary mucinous adenocarcinoma whose HRCT is similar to diffuse pneumonia, with a mild clinical symptoms. This may explain the fact that a part of the DPLDs is tumors, and the 100% diagnosis of mucinous adenocarcinomas. Cortese et al. reported that bronchoscopic biopsy of very localized lesions yields a diagnostic rate of 60% in primary lung cancer and 50% in metastatic cancer,^27^ which is not completely inconsistent with our results. The reason for these inconsistencies may be the differences in our patient selection, and our study's exclusion of biopsy of peripheral nodules and lesions originating in the bronchial wall.

The current study is limited by its retrospective nature and the difficulty in determining how results from TBLB were used. The present study attempted to follow strict criteria for definitions of usefulness or unusefulness; however, a degree of subjectivity had been necessary in some cases. We attempted to focus on non‐infectious DPLD; thus, the diagnostic role of TBLB in DPLD as a whole could not be reflected. Most of the specific histological diagnoses were CTD‐ILDs; however, the design of this study precluded speculation regarding the specificity of the primary disease patterns. Moreover, TBLC is currently an emerging technique in the diagnosis of DPLD. Nevertheless, in our hospital, TBLC was not routinely used in the diagnosis of diffuse lung disorders, with the main reasons being pneumothorax and bleeding adverse events. Ravaglia et al. reported that 0.4% of cases died within 30 days after TBLC.^2^ However, another study compared the yield of TBLB and TBLC and the patients underwent flexible bronchoscopy with TBLB followed by TBLC. This study concluded that TBLC used with TBLB can improve the diagnostic yield of flexible bronchoscopy in patients with DPLD.^28^ In our hospital alone, >2000 bronchoscopies are performed each year, and about 470 patients undergo TBLB. These figures reflect our clinical practice in vivo. In this study, TBLB performance yielded a lower incidence of pneumothorax and bleeding, and we found that the technique is very safe in daily clinical practice, even without fluoroscopy guidance. As a result, bronchoscopy remains an important diagnostic technique for the evaluation of patients with DPLD.

## 5 CONCLUSIONS

In summary, we found that TBLB served as a key determinant or as supplementary information in the final diagnosis of non‐infectious DPLDs. TBLB decision‐making should be based on clinical and radiological data. Nevertheless, future studies may require multiple methods in the diagnosis of DPLD.

## CONFLICT OF INTEREST

This study has no potential conflict of interest with any institution or individual. The authors declare that they have no competing interests.

## ETHICS STATEMENT

This study was reviewed and approved by the ethics committee of Capital Medical University. All patients provided written informed consent before undergoing bronchoscopy.

## AUTHOR CONTRIBUTIONS

Lefei Zhou designed the idea of the article and wrote the manuscript together with Feng Wang; Xiaoguang Xu, Lili Xu, and Zhen Wang collected and compiled the clinical data needed for this article; Lefei Zhou and Feng Wang conducted statistics and analysis of the results. Finally, Zhaohui Tong revised and corrected the manuscript.

## Data Availability

Not applicable.
